# Reply to comment on Peto *et al*. (2020): Weekly COVID-19 testing with household quarantine and contact tracing is feasible and would probably end the epidemic

**DOI:** 10.1098/rsos.210467

**Published:** 2021-06-23

**Authors:** Julian Peto

**Affiliations:** London School of Hygiene and Tropical Medicine, London, UK

An epidemic model is a hypothesis, not an observation. Few scientists would question our statement that the impact of ‘the combination of weekly SARS-CoV-2 testing with an earlier test if symptoms appear, strict household quarantine and contact tracing … cannot be reliably predicted by further modelling’ [1, p. 3]; yet Planck and colleagues [2] claim that their simple model shows that mass weekly testing and household quarantine, even if it were perfectly achievable, would not be sufficient to control the spread of COVID-19. This is contradicted by the transient reversal of rising prevalence in Slovakia after two rounds of weekly national testing and household quarantine. Prevalence fell by 58% within a week, and a microsimulation calibrated to the observed results confirms that quarantining the whole household following a positive test made a dominant contribution, with an estimated weekly reduction in the prevalence of 59% with and only 10% without household quarantine [3]. We need real data on the effects of different population testing protocols in whole cities [4], not uncalibrated simulations predicting that it cannot work.
